# Publisher Correction: Large language models encode clinical knowledge

**DOI:** 10.1038/s41586-023-06455-0

**Published:** 2023-07-27

**Authors:** Karan Singhal, Shekoofeh Azizi, Tao Tu, S. Sara Mahdavi, Jason Wei, Hyung Won Chung, Nathan Scales, Ajay Tanwani, Heather Cole-Lewis, Stephen Pfohl, Perry Payne, Martin Seneviratne, Paul Gamble, Chris Kelly, Abubakr Babiker, Nathanael Schärli, Aakanksha Chowdhery, Philip Mansfield, Dina Demner-Fushman, Blaise Agüera y Arcas, Dale Webster, Greg S. Corrado, Yossi Matias, Katherine Chou, Juraj Gottweis, Nenad Tomasev, Yun Liu, Alvin Rajkomar, Joelle Barral, Christopher Semturs, Alan Karthikesalingam, Vivek Natarajan

**Affiliations:** 1 Google Research, Mountain View, CA USA; 2 National Library of Medicine, Bethesda, MD USA; 3 DeepMind, London, UK

**Keywords:** Health care, Medical research

Correction to: *Nature* 10.1038/s41586-023-06291-2 Published online 12 July 2023

In the version of this article initially published, refs. 1–14 were disordered due to a conversion error; the reference numbers are now amended in the HTML and PDF versions of the article.

