# Supplementary material for: Large language models encode clinical knowledge
Source: Nature. 2023 Jul 12;620(7972):172–80. doi: 10.1038/s41586-023-06291-2 (PMC10396962; doi:10.1038/s41586-023-06291-2)
Supplement: Supplementary file 2 — Reporting Summary [file 41586_2023_6291_MOESM2_ESM.pdf]

nature portfolio

Corresponding author(s): Karan Singhal
Shekoofeh Azizi
Alan Karthikesalingam
Vivek Natarajan

Last updated by author(s): April 18, 2023

Reporting Summary

Nature Portfolio wishes to improve the reproducibility of the work that we publish. This form provides structure for consistency and transparency in reporting. For further information on Nature Portfolio policies, see our Editorial Policies and the Editorial Policy Checklist.

Statistics

For all statistical analyses, confirm that the following items are present in the figure legend, table legend, main text, or Methods section.

n/a	Confirmed	
☐	☒	The exact sample size (*n*) for each experimental group/condition, given as a discrete number and unit of measurement
☐	☒	A statement on whether measurements were taken from distinct samples or whether the same sample was measured repeatedly
☐	☒	The statistical test(s) used AND whether they are one- or two-sided<br>*Only common tests should be described solely by name; describe more complex techniques in the Methods section.*
☐	☒	A description of all covariates tested
☒	☐	A description of any assumptions or corrections, such as tests of normality and adjustment for multiple comparisons
☐	☒	A full description of the statistical parameters including central tendency (e.g. means) or other basic estimates (e.g. regression coefficient) AND variation (e.g. standard deviation) or associated estimates of uncertainty (e.g. confidence intervals)
☐	☒	For null hypothesis testing, the test statistic (e.g. *F*, *t*, *r*) with confidence intervals, effect sizes, degrees of freedom and *P* value noted<br>*Give P values as exact values whenever suitable.*
☒	☐	For Bayesian analysis, information on the choice of priors and Markov chain Monte Carlo settings
☒	☐	For hierarchical and complex designs, identification of the appropriate level for tests and full reporting of outcomes
☒	☐	Estimates of effect sizes (e.g. Cohen's *d*, Pearson's *r*), indicating how they were calculated

Our web collection on statistics for biologists contains articles on many of the points above.

Software and code

Policy information about availability of computer code

Data collection	The study used six open source medical question answering for which no software was required. The additional open source dataset we plan to release as part of the study, HealthSearchQA, required scripts in python 3.10 for curation.
Data analysis	We will not be able to open source the large language models (LLMs) used in this study. We have provided comprehensive details regarding our underlying methodology. We have also released related models at https://huggingface.co/google/flan-t5-xl

For manuscripts utilizing custom algorithms or software that are central to the research but not yet described in published literature, software must be made available to editors and reviewers. We strongly encourage code deposition in a community repository (e.g. GitHub). See the Nature Portfolio guidelines for submitting code & software for further information.

Data

Policy information about availability of data

All manuscripts must include a data availability statement. This statement should provide the following information, where applicable:

- Accession codes, unique identifiers, or web links for publicly available datasets
- A description of any restrictions on data availability
- For clinical datasets or third party data, please ensure that the statement adheres to our policy

The benchmark used in the study, MultiMedQA, comprises six open source datasets and an additional one on consumer medical questions, HealthSearchQA, which we newly introduce. HealthSearchQA dataset is provided as a supplementary file. MedQA - https://github.com/jind11/MedQA, MedMCQA - https://medmcqa.github.io, PubMedQA - https://pubmedqa.github.io, LiveQA - https://github.com/abachaa/LiveQA_MedicalTask_TREC2017, MedicationQA - https://github.com/abachaa/Medication_QA_MedInfo2019, MMLU - https://huggingface.co/datasets/hendrycks_test

Human research participants

Policy information about studies involving human research participants and Sex and Gender in Research.

Reporting on sex and gender	N/A
Population characteristics	N/A
Recruitment	N/A
Ethics oversight	N/A

Note that full information on the approval of the study protocol must also be provided in the manuscript.

Field-specific reporting

Please select the one below that is the best fit for your research. If you are not sure, read the appropriate sections before making your selection.

☒ Life sciences ☐ Behavioural & social sciences ☐ Ecological, evolutionary & environmental sciences

For a reference copy of the document with all sections, see nature.com/documents/nr-reporting-summary-flat.pdf

Life sciences study design

All studies must disclose on these points even when the disclosure is negative.

Sample size	The majority of datasets used in the study are already open source and have been used in the community for several years. As such, they have proven sufficient to estimate model performance accurately. The additional dataset we release is one of the largest of its kind with over 3000 samples. For the human evaluation, we chose 140 questions. A specific sample size calculation was not done.
Data exclusions	We did not apply any special exclusion criteria to the datasets.
Replication	We have repeated our experiments independently three times to confirm the accuracy of the results for the MedQA dataset. The variance was minimal as detailed in the paper.
Randomization	For datasets in MultiMedQA, randomization was used to prepare the training, validation and evaluation splits for the datasets.
Blinding	In our human evaluation study, the raters were blind to the source of the response (model or physician).

Reporting for specific materials, systems and methods

We require information from authors about some types of materials, experimental systems and methods used in many studies. Here, indicate whether each material, system or method listed is relevant to your study. If you are not sure if a list item applies to your research, read the appropriate section before selecting a response.

Materials & experimental systems

n/a	Involved in the study	
☒	☐	Antibodies
☒	☐	Eukaryotic cell lines
☒	☐	Palaeontology and archaeology
☒	☐	Animals and other organisms
☒	☐	Clinical data
☒	☐	Dual use research of concern

Methods

n/a	Involved in the study	
☒	☐	ChIP-seq
☒	☐	Flow cytometry
☒	☐	MRI-based neuroimaging
