# Supplementary figures and images for: Large language models encode clinical knowledge
Source: Nature. 2023 Jul 12;620(7972):172–80. doi: 10.1038/s41586-023-06291-2 (PMC10396962; doi:10.1038/s41586-023-06291-2)

MedQA

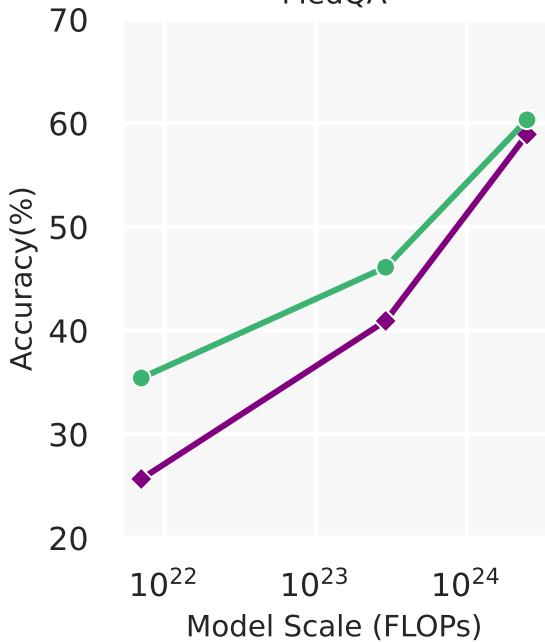

MedMCQA

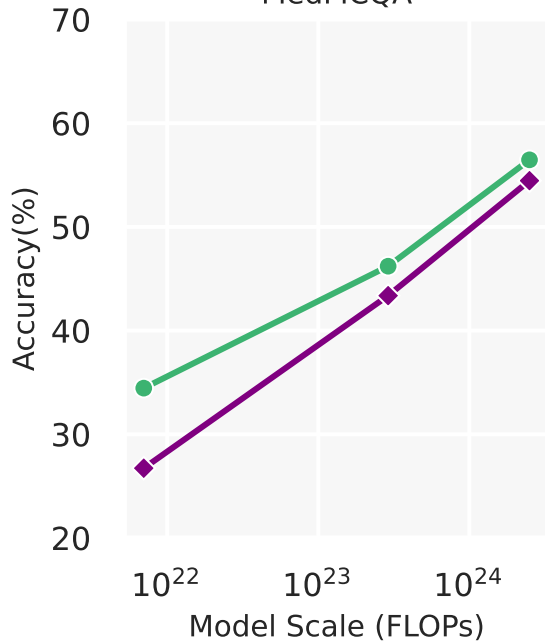

PaLM Few Shot

Flan-PaLM Few Shot

Supplement: Supplementary file 4 — Scaling plots for PaLM and Flan-PaLM with few-shot prompting on MedQA and MedMCQA. [file 41586_2023_6291_MOESM4_ESM.pdf]

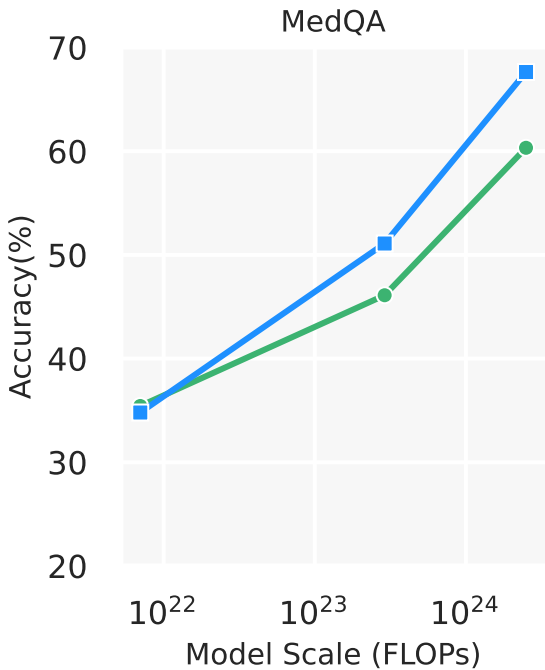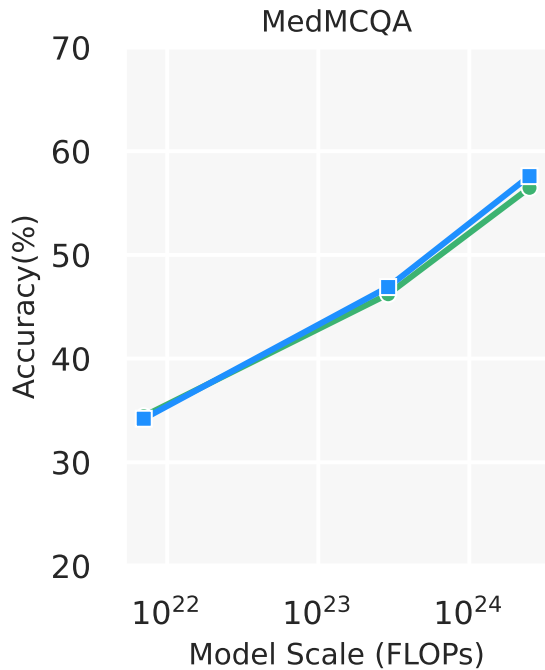

— Flan-PaLM Few Shot      — Flan-PaLM Few Shot+COT+SC

Supplement: Supplementary file 5 — Scaling plots for Flan-PaLM with few-shot and Flan-PaLM few-shot + COT + self-consistency on MedQA and MedMCQA. [file 41586_2023_6291_MOESM5_ESM.pdf]
